# Determination of Clinical Parameters Sensitive to Functional Voice Disorders Applying Boosted Decision Stumps

**DOI:** 10.1109/JTEHM.2020.2985026

**Published:** 2020-05-22

**Authors:** Patrick Schlegel, Andreas M. Kist, Marion Semmler, Michael Döllinger, Melda Kunduk, Stephan Dürr, Anne Schützenberger

**Affiliations:** 1 Department of Otorhinolaryngology Head and Neck SurgeryDivision of Phoniatrics and Pediatric AudiologyUniversity Hospital Erlangen, Friedrich-Alexander-University Erlangen-Nürnberg91054ErlangenGermany; 2 Department of Communication Sciences and DisordersLouisiana State University 5779 Baton RougeLA70803USA

**Keywords:** Parameters, boosted decision Stumps, classification, functional dysphonia

## Abstract

Background: Various voice assessment tools, such as questionnaires and aerodynamic voice characteristics, can be used to assess vocal function of individuals. However, not much is known about the best combinations of these parameters in identification of functional dysphonia in clinical settings. Methods: This study investigated six scores from clinically commonly used questionnaires and seven acoustic parameters. 514 females and 277 males were analyzed. The subjects were divided into three groups: one healthy group (N_01_) (49 females, 50 males) and two disordered groups with perceptually hoarse (FD_23_) (220 females, 96 males) and perceptually not hoarse (FD_01_) (245 females, 131 males) sounding voices. A tree stumps Adaboost approach was applied to find the subset of parameters that best separates the groups. Subsequently, it was determined if this parameter subset reflects treatment outcome for 120 female and 51 male patients by pairwise pre- and post-treatment comparisons of parameters. Results: The questionnaire “Voice-related-quality-of-Life” and three objective parameters (“maximum fundamental frequency”, “maximum Intensity” and “Jitter Percent”) were sufficient to separate the groups (accuracy ranging from 0.690 (FD_01_ vs. FD_23_, females) to 0.961 (N_01_ vs. FD_23_, females)). Our study suggests that a reduced parameter subset (4 out of 13) is sufficient to separate these three groups. All parameters reflected treatment outcome for patients with hoarse voices, Voice-related-quality-of-Life showed improvement for the not hoarse group (FD_01_). Conclusion: Results show that single parameters are insufficient to separate voice disorders but a set of several well-chosen parameters is. These findings will help to optimize and reduce clinical assessment time.

## Graphical abstract

In this study, we show that only a small set of four parameters is sufficient to differentiate between healthy subjects and patients with diagnosis of functional dysphonia. These four parameters also reflect treatment outcome for functional dysphonia and perceivable hoarse voices (success or failure). Thus, in future a functioning set of objective tools could be created that improves and accelerates assessment and therapy of voice disorders.

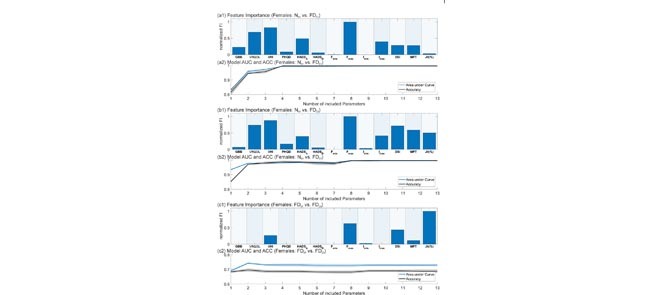

## I. Introduction

An overwhelming amount of clinical workload with too little time per patient is a general problem for physicians, as variously implied [1]–[3]. Apart from the obvious effects on the work-life balance of clinicians, it was found that high workload affects quality of teaching [4], as well as quality of treatment. In one study, over 20% of partaking hospitalists reported an influence of their workload to patient transfers, morbidity and mortality [1]. Therefore it will be beneficial to both for clinicians and patients to reduce the amount of paperwork and workload they have to go through during diagnostic and treatment procedures in a clinic setting. The field of laryngology is no exception from this situation [5], [6].

In laryngology, among others, the voice generating process enabling for phonation, articulation and speech is of interest. Phonation is achieved by the airstream, rising from the lungs, setting the vocal folds, vibratory sound source of the phonation [7], in motion. The vocal folds begin to oscillate, periodically interrupting the airflow and generating audible sound [8], [9]. In literature, a variety of different frequency ranges for normal vocal fold oscillations is given with upper boundaries from about 250 Hz [10] to 400 Hz [11]. For the female singing voice even frequencies as high as 1568 Hz for vocal fold oscillations with complete closure were reported [12]. After passing the vocal folds, the airflow and thereby the sound is further modulated by the vocal tract, tongue and lips [8], [9].

A symmetric and periodic vocal fold oscillation pattern with regular glottal closure is usually associated with a healthy voice [13]–[15]. Respectively, aperiodic and asymmetric oscillations of the vocal folds are usually associated with disordered voice, even in absence of other structural or neurologic impairments [16]–[18]. However, newer investigations by Semmler *et al.* indicate that this “symmetric equals healthy” equation may not be applicable on vertical vibration components [19]. In the absence of structural or neurological impairments a voice disorder is classified under the broad term “functional dysphonia” (FD) [16].

Symptoms of FD may include changes in pitch, loudness, fatigue and other changes of voice quality but not have to include hoarseness. Since FD is basically diagnosed by excluding other (organic) causes of a voice disorder, underlying causes of FD may vary; e.g. emotional and pure psychological factors for voice impairment are in the range of possibilities [11], [20], [21]. Hence, FD does not necessarily mean an audible hoarse voice. This means that some people suffering from FD may have a hoarse voice and therefore an acoustically measurable symptom. The improvement of voice quality can be assessed after treatment. Other subjects may report only hardly, if at all, audible assessable symptoms. Although diagnosis and treatment is similar, it is reasonable to differentiate between both groups i.e. subjects with FD and perceivable hoarse voices and subjects with FD and without perceivable hoarse voices. This is also confirmed by our results showing significantly different behavior of parameters for both groups during treatment.

Different rating systems exist to grade the hoarseness degree of patients. Internationally three prominent systems are in use: the GRBAS scale, Cape-V and the roughness-breathiness-hoarseness voice perceptual evaluation system (RBH). In German speaking countries, the latter is widely applied [22]. This system is based on the assessment of the roughness (R) and the breathiness (B) of a voice on a 0 to 3 scale by a clinician with 0 indicating no impairment and 3 indicating maximum roughness/breathiness. Subsequently the hoarseness H (RBH-H) is determined as the maximum of the first two values R and B and is in the following used to differentiate between groups [22]–[25].

Different approaches have been made to separate healthy and disordered voices [26]–[28]. Awan and Roy investigated various time and spectral-based acoustic measures resulting in a 5-variable model that correctly classified voice type (normal, breathy, hoarse or rough) with 0.75 accuracy [26]. Callan *et al.* achieved an accuracy of 0.76 using a self-organizing map on acoustic measures for the classification of normal and disordered female voices [27]. Voigt *et al.* classified functional voice disorders based on phonovibrograms with an accuracy of 0.81 [28]. However, all these results were achieved for rather small datasets ranging from 75 to 134 subjects [26]–[28].

Voice assessment has different tools to investigate different parts of voice production, such as high-speed videoendoscopy (HSV) for recording the fast oscillations of the vocal folds [29] or audio analysis systems, such as Praat [30]. Whilst tools as HSV are primarily important for diagnostics [11], a growing amount of protocols exists for the treatment of FD [31], [32]. New therapy approaches are proposed and investigated regularly [33]–[35]. Hereby, a great variety of questionnaires and objective parameters were proposed [36]–[38] and studies were conducted to evaluate the performance of questionnaires [39], [40].

Clinicians have a wide range of assessment tools that can be used to ensure accurate diagnosis of voice disorders to initiate effective and precise treatment techniques for the voice disorder. Therefore, the idea of questionnaires and objective acoustic and endoscopic parameters is to get a detailed assessment and determine the treatment outcome of voice disorders. However, many parameters are not well-understood, mathematical dependencies between parameters and influences affecting parameters exist [41], [42] and it is not clear which parameters are the most reliable ones. Therefore, the creation of a standardized set of parameters is necessary so that: (1) clinical workload is reduced (2) medical professionals from different institutions can assess patient histories without communication problems (3) diagnostics can be quantified for e.g. health insurance reasons (4) patients can better estimate the progress of their treatment (5) clinicians can better judge treatment outcome and progress.

However, up to now, there have been limited attempts investigating the explanatory power of parameters in actual clinical environment. Hence, it is not clear which parameters are the most effective ones, and how many of them are needed for differentiating between different voice types. To get closer to answering these questions, this study investigates six scores from commonly applied self-assessment questionnaires and seven objective parameters using a large quantity of clinical data collected in a period of more than three years. Healthy subjects as well as patients diagnosed with FD with hoarse sounding voices and FD with not hoarse sounding voices (but other non-organic voice problems as i.e. high voice effort, voice changes or psychologically caused problems) were examined. The aims of this work were:

1) Determine which parameters can differentiate best between three different groups; healthy group (N_01_), perceptually hoarse (FD_23_) and perceptually not hoarse (FD_01_)
2) Find a minimal subset of parameters that can be used to separate all groups.
3) Ascertain if the found parameters improve during treatment; i.e., compare pre- post treatment status.

## II. Methods

In total 514 females and 277 males were investigated. The females and males were divided into three groups each: a healthy group with normal sounding voices (N_01_) and two disordered pre-treatment groups. Both pre-treatment groups consisted of patients with diagnosed FD. However, the first pre-treatment group (FD_01_) had not hoarse voices, as judged by our clinicians. This is indicated by the clinicians giving the voices of the patients a low RBH-H rating of 0 or 1. This group included patients with different not-hoarseness related voice problems (e.g. high effort to speak [16], [43]). Group three includes patients with hoarse voices and their voice judged perceptually by a high RBH-H rating of 2 or 3 (FD_23_). The subjects were separated this way since perceptually not hoarse voices generally received an RBH-H rating of 0 or 1. Hoarse voices are mainly rated 2 and in few cases even with 3. Therefore, only two voice disordered pre-treatment groups were formed using the four-level RBH scale.

In Table 1, the three groups for females and males are listed along with the number of subjects that were assigned to them. Furthermore, the numbers of subjects in groups FD_01_ and FD_23_ having post-treatment exams are listed. Post-treatment patients were examined between one week and one year after treatment forming three post-treatment groups, respectively separated for females and males:

1) perceived not hoarse before and after treatment (**FD**_**01**_/**PT**_**01**_)
2) perceived hoarse before but not after treatment (**FD**_**23**_/**PT**_**01**_)
3) perceived hoarse before and after treatment (**FD**_**23**_/**PT**_**23**_)

**TABLE 1:** Pre- and Post-Treatment Groups

Healthy and Pre-Treatment	$\text{N}_{{01}}$	FD_01_	FD_23_
Females	49	245	220
Males	50	131	96
Post-Treatment	FD_01_/PT_01_	FD_23_/PT_01_	FD_23_/PT_23_
Females	46	42	32
Males	29	12	10

Number of patients in healthy, pre- and post-treatment groups (one healthy, two pre- and two post-treatment groups) included in this study. The different post-treatment groups contain patients with consistently low RBH-H (FD_01_/PT_01_), patients with RBH-H decreasing from pre- to post-treatment (FD_23_/PT_01_) and patients with consistently high RBH-H (FD_23_/PT_23_).

Table 2 shows all 13 parameters that were investigated in this study, their abbreviations, a short explanation, percentage of missing data values, the value range and which values indicate a healthy/normal voice. Parameters were chosen based on recommendations in the ELS-protocol [31] and are collected during our daily clinical routine. Also not directly voice-related questionnaires (GBB, PHQD, HADS_A_, HADS$_{\mathrm {D}}$) are included, since for some patients suffering e.g. from depression, FD may only be the secondary disease. In total the scores of six commonly used self-assessment questionnaires and seven objective parameters were investigated as they are collected during our daily clinical working routine. German questionnaires [44] or German versions of internationally applied questionnaires [36], [40], [45], [39] were used. The classification-parameter, RBH-H, was used to differentiate between hoarse and not hoarse sounding voices in the disordered groups. The inter-rater reliability of RBH-H was investigated in a previous study based on running speech. The study investigated 78 voice samples rated by 19 different logopedic students resulting in a Cronbach alpha of 0.924 for RBH-H values [46].

**TABLE 2:** Parameter Information

Parameter and reference	Abbreviation	Parameter description	% missing	range
self-assessment questionnaires				
Gießener Beschwerdebogen 24 [44]	GBB	Questionnaire score that assesses the general impairment of life quality.	56.6%	0-96
Voice-Related-Quality-of-Life [36]	VRQOL	Questionnaire score that assesses the impairment of life quality by voice.	14.4%	0-100
Voice Handicap Index [39]	VHI	Questionnaire score that assesses the impairment of life quality by voice.	48.2%	0-120
Patient Health Questionnaire (short form) [40]	PHQD	Questionnaire score that assesses anxiety- and depression-related symptoms.	45.8%	0-32
Hospital Anxiety and Depression Scale (anxiety partition) [45]	HADS_A_	Score of a section of a questionnaire that assesses anxiety-related symptoms	54.1%	0-21
Hospital Anxiety and Depression Scale (depression partition) [45]	HADS_D_	Score of a section of a questionnaire that assesses depression-related symptoms.	54.3%	0-21
objective parameters(measured during examination)				
Minimum fundamental frequency	$\text{F}_{\mathrm {min}}$	Lowest fundamental frequency that the subject is able to produce.	3.2%	Low^*^
Maximum fundamental frequency	$\text{F}_{\mathrm {max}}$	Highest fundamental frequency that the subject is able to produce.	2.8%	High
Lowest generatable sound intensity	$\text{I}_{\mathrm {min}}$	Lowest vocal sound intensity that the subject is able to produce.	2.8%	Low
Loudest generatable sound intensity	$\text{I}_{\mathrm {max}}$	Highest vocal sound intensity that the subject is able to produce.	3.2%	High
Maximum phonation time	MPT	Maximum time the subject can hold sustained phonation.	10.7%	High
objective parameters(calculated from audio segments)				
Dysphonia Severity Index [37]	DSI	Objective measure that is calculated as a weighted sum of $\text{F}_{\mathrm {max}}$, $\text{I}_{\mathrm {min}}$, MPT and Jit(%)	10.6%	High
Jitter (%) [38]	Jit(%)	Normalized period perturbation measure.	10.6%	Low
parameter used for classification				
Roughness breathiness hoarseness system, hoarseness partition [22]	RBH-H	External assessment measure of voice quality, rated by clinicians.	0%	0-3

Parameter information containing parameter names, abbreviations, a short description and the value ranges of the parameters with bold print marking the end of the range that indicates healthy voices.

^*^ for some parameters no valid range could be given, as no exact limits for the ranges of these parameters are defined. In these cases it was merely noted if ”low” or ”high” values indicate a healthy voice.

Objective parameters were calculated using the software tool Lingwaves by Wevosys with default settings for audio segments of sustained /a/ vowels. All audio segments were between three to five seconds long. The maximum phonation time was measured separately for each subject. Maximum and minimum frequency and volume were also measured separately by asking the subjects to phonate as high/low, loud/soft as possible. All other calculations in this study were performed using custom-written software in MATLAB (version 9.3.0.713579, R2017b). The study was approved by the ethic committee of the Medical School at the Friedrich-Alexander-University Erlangen-Nürnberg (no. 290_13B).

### A. Influence of Subject Age

**FIGURE 1.**
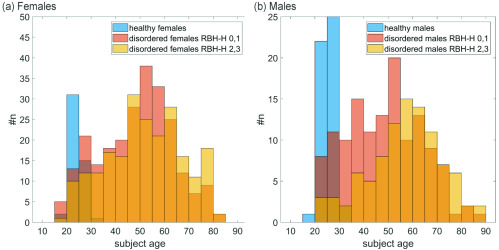
Distribution of subject age for (a) females and (b) males for healthy and pre-treatment groups with #n being the number of subjects.

In Fig. 1, the age distribution of the healthy subjects and all disordered subjects for females and males is shown. The great difference in age between the healthy and the disordered group is a common problem in clinical studies [47], [48]. To ensure that the influence of subject age on the results of this study is negligible, we performed the following analysis for both, disordered pre-treatment groups of females and males:

First, we calculated the Spearman correlation coefficient (CC) between each of the 13 parameters and the age of the subjects as well as the p-value of the correlation. This p-value states if the correlation is statistically significantly different from zero for a significance level alpha of 0.05. Mukaka published a frequently cited work regarding the correct use of correlation coefficients in medical research also containing a table providing a “rule of thumb” for the interpretation of correlation coefficients. According to this table, correlations below 0.3 are negligible and correlations between 0.3 and 0.5 are seen as low [49]. In this study only five CCs between 0.3 and 0.5 were detected, hence the overall influence of age on all parameters was judged as not critical. For more detailed information see Results, section “Correlation coefficients between parameters and age”.

### B. Model Selection and Optimization

For finding the set of parameters that can differentiate best between the groups N_01_, FD_01_ and FD_23_, we generated models for class/group separation. Each separation task between two of the classes is one model. To train those models in separating the classes, we chose to use the supervised learning classification approach of single level boosted trees (also known as boosted stumps) [50].

A stump or decision stump is the shortest possible form of a decision tree, consisting only of one node and two leaves. Each model consists of multiple of these stumps that work sequentially with the data applying a boosting approach. The use of trees allows for a partly compensation of missing values by the use of surrogate splits [51], [52]. Surrogate splits were preferred over data augmentation since a total of 24% of all data entries were missing. Therefore extensive data augmentation would have introduced considerable data distortions.

In a comprehensive study comparing a great number of different classification algorithms, boosted stumps achieved high scores of correctly classified samples for a range of different datasets and measures of classifier performance. Boosted stumps sometimes even outperformed the overall best boosted trees classification method [53]. In our study we decided to use boosted stumps instead of fully grown boosted trees to also avoid overfitting [51]. To find parameters that differentiate best the three classes, we performed three different group comparisons for males and females:

1) N_01_ vs. FD_01_
2) N_01_ vs. FD_23_
3) FD_01_ vs. FD_23_

Four different boosted trees algorithms were investigated using the MATLAB function “fitcensemble”, namely AdaBoost, LogitBoost, GentleBoost and RUSBoost. We included AdaBoost as it is one of the most popular algorithms in this field [51] and hence a common choice. Each of the other algorithms is designed to handle one of different special cases. However, all of these cases apply to our data. LogitBoost is designed for hardly separable classes, GentleBoost for multilevel categorical predictors and RUSBoost for imbalanced class sizes (see also [54]). The function “fitcensemble” allows the use of different so called “name value pair arguments” to account for characteristics of diverse types of datasets. The following Name value pair arguments were used in this study:

1) ‘prior’ was set to ‘uniform’ because of imbalanced class sizes,
2) ‘surrogate’ was set to ‘on’ to be able to factor in data rows with missing values,
3) ‘MaxNumSplits’ was set to 1 to avoid overfitting (i.e. all trees consisted out of only one node),
4) ‘LearningRate’ was set to 0.1 for training with shrinkage to find a better optimum.

The performance of all algorithms was rated based on **two factors**. The **first factor** was “Area Under Curve” (AUC) and the “Accuracy” (ACC) an algorithm achieved in separating the subject groups (the higher the better). Since one drawback of ACC is that it can produce misleading results for unbalanced class sizes we also included the performance measure AUC. AUC measures the discriminatory power between classes and is immune to unbalanced class sizes but can be misleadingly low for extremely sharply separated classes. However, in such cases ACC can be a useful additionally measure, hence both measures complement each other to some degree.

Nevertheless, in case of low ACC and AUC sensitivity (share of true positives in the data) and specificity (share of true negatives in the data) were calculated to ensure that not one class was overly preferred during classification (i.e. high specificity and low sensitivity or vice versa). However, since models are recalculated ten times, only the averaged absolute difference between sensitivity and specificity is given. If sensitivity and specificity were given separately, it could result in both values being misleadingly close. This would e.g. be the case, if, for some model evaluations, sensitivity is higher than specificity and vice versa for other model evaluations. For more details on performance measures see [51].

The **second factor** analyses how much the four boosted tree algorithms weight a set of four added random parameters by feature importance (FI) (the lower the better). FI is a measure that states how important single features (i.e. parameters) are for the correct classification of subjects by the algorithm [55]. The added random parameters are a normally-distributed variable, a normally-distributed variable with 50% missing numbers, an equally distributed variable and an equally distributed variable with 50% missing numbers. Ten-fold cross validation was used, and AUC and ACC values were calculated on the respective validation i.e. testing partition of the datasets. This and all following evaluation steps involving cross validation were repeated ten times for each group comparison and only the averages of AUC and ACC values were investigated. We did this to reduce the effect of random partitioning on the results of this study.

The algorithm achieving best AUC and ACC ratings of the four tested algorithms was AdaBoost. To assure that the random parameters did not affect this outcome, the models were rebuilt without the random parameters and AdaBoost still achieved best AUC and ACC. Furthermore, AdaBoost also rated the random parameters with low importance. For this algorithm each model was optimized using the AUC. For each group comparison the AUC was calculated for cross-validated models (without random parameters) consisting of only one tree stump up to models consisting of 500 consecutive tree stumps (i.e. models of increasing degree of complexity). Thereby the optimal number of stumps yielding the best results was determined.

After the best model with the optimal number of tree stumps (which was 300) was determined, the FI for those models containing all 13 parameters (this time without the random parameters) for all group comparisons was calculated. For each group comparison the parameters were sorted by FI. Afterwards AUC and ACC were calculated for further models only containing one parameter (the parameter that obtained the highest FI), two parameters (the parameter that obtained the highest and the parameter that obtained the second highest FI) until all 13 parameters were included in the last model. Based on this, a number of parameters was selected, that still provided a high ACC and AUC excluding the less informative parameters and hence determining the relevant parameters for each group comparison. A final optimal set of four parameters was proposed that was able to separate all three group comparisons most efficiently (for details see Results).

### C. Comparing Pre- and Post-Treatment Groups

It was investigated if the four found parameters for females and males reflect the outcome of treatment. Therefore subjects from pre-treatment groups (FD_01_, FD_23_) and post-treatment groups (FD_01_/PT_01_, FD_23_/PT_01_, FD_23_/PT_23_) were compared (pairwise comparisons). The following three pre-post-treatment-comparisons were investigated separated for females and males:

1) Pre-treatment with RBH-H = 0,1 vs. post-treatment with RBH-H = 0,1 (PrePost_01/01_)
2) Pre-treatment with RBH-H = 2,3 vs. post-treatment with RBH-H = 0,1 (PrePost_23/01_)
3) Pre-treatment with RBH-H = 2,3 vs. post-treatment with RBH-H = 2,3 (PrePost_23/23_)

For the different comparisons, e.g. PrePost_23/01_, only subjects were considered that were part of both groups (FD_23_ and FD_23_/PT_01_), due to pairwise/dependent comparisons. This analogously applies to the other pre-post-treatment comparisons. The change of RBH-H between pre- and post-treatment groups was used as an indicator for no hoarseness related change (continuously RBH-H = 0,1), treatment success (improved RBH-H) or treatment failure (continuously RBH-H = 2,3). Pairwise one-sided Wilcoxon tests were conducted with the H_0_ hypothesis that the parameter value stayed the same or worsened after treatment. Depending on the parameter a “worsening” can mean an increase or decrease in value. In Table 2 is mentioned for each parameter if high or low values indicate a disordered condition. Since multiple groups were tested, Bonferroni correction was applied for each of the pre-post-treatment-comparisons resulting in a rejection of H0 only if the p-value was less than 0.05/3~0.017.

## III. Results

### A. Summary

Statistical analysis revealed an overall low and neglectable influence of subject age. VRQOL, $\text{I}_{\mathrm {max}}$, $\text{F}_{\mathrm {max}}$ and Jit(%) were found to be the most reliable parameter subset for differentiating between groups N_01_, FD_01_ and FD_23_. Furthermore those parameters also reflected changes between pre- and post-treatment groups. Table 3 contains mean AUC and ACC for all group comparisons for the original set of parameters and the reduced set as well as the average of AUC and ACC over all group comparisons. Due to missing values in the data, the numbers of available data values are given in all tables. The average AUC of the model using only four parameters was over all group comparisons only 0.018 less than the AUC of the model using all parameters. Similarly, for ACC the difference was 0.033. Therefore the average performance of the models including all parameters is only marginally better than the performance of the models including only the parameters VRQOL, $\text{I}_{\mathrm {max}}$, $\text{F}_{\mathrm {max}}$ and Jit(%).

**TABLE 3:** Comparison of Full and Reduced Model

Group comparison	AUC	ACC
Females: Reduced set (VRQOL, $\text{I}_{\mathrm {max}}$, $\text{F}_{\mathrm {max}}$ and Jit(%)) vs. Complete set: (13 parameters)		
N_01_ vs. FD_01_ #49 vs. #245	0.955 vs. 0.995	0.930 vs. 0.997
N_01_ vs. FD_23_ #49 vs. #220	0.977 vs. 0.997	0.961 vs. 0.996
FD_01_ vs. FD_23_ #245 vs. #220	0.734 vs. 0.729	0.690 vs. 0.687
Males: Reduced set (VRQOL, $\text{I}_{\mathrm {max}}$, $\text{F}_{\mathrm {max}}$ and Jit(%)) vs. Complete set: (13 parameters)		
N_01_ vs. FD_01_ #50 vs. 131	0.965 vs. 0.999	0.915 vs. 0.988
N_01_ vs. FD_23_ #50 vs. 96	0.992 vs. 0.998	0.958 vs. 0.979
FD_01_ vs. FD_23_ #131 vs. 96	0.734 vs. 0.749	0.714 vs. 0.718
Average over all group comparisons		
Reduced set vs. Complete set	0.893 vs. 0.911	0.861 vs. 0.894

Comparison of mean AUC and ACC (averaged over all repartitioned models) between models trained with all parameters and models trained only with the reduced set of parameters. ”#x” vs. ”#y” denotes the numbers of available samples per group.

In Table 4 the median values and the median absolute deviation of VRQOL, $\text{I}_{\mathrm {max}}$, $\text{F}_{\mathrm {max}}$ and Jit(%) as well as the number of samples as range for all groups are summarized. The median absolute deviation is a measure of dispersion that calculates the median distance between the median of a data vector and all of its entries (i.e. the “standard deviation” for ordinal scaled data).

**TABLE 4:** Values for Healthy and Pre-Treatment Groups

	VRQOL	$\text{I}_{\mathrm {max}}$	$\text{F}_{\mathrm {max}}$	Jit(%)
Females median/median absolute deviation				
N_01_ #48–49	100/0	98.5/4.5	919/184	0.145/0.04
FD_01_ #210–242	70/15	90/5.5	551/118	0.200/0.08
FD_23_ #178–207	57/17	85/6	437/101	0.380/0.23
Males median/median absolute deviation				
N_01_ #49–50	100/0	97.5/6.5	686/116.5	0.09/0.02
FD_01_ #106–129	77/15	92/5	366/89	0.130/0.06
FD_23_ #74–93	67/15	87.5/5.5	330/97	0.290/0.20

Median and median absolute deviation of all healthy and pre-treatment groups. The range ”#x-#y” denotes the number of available samples that were minimally and maximally available for the parameters.

### B. Correlation Coefficients Between Parameters and Age

Table 5 lists the calculated correlation coefficients (CC)s for males and females for each of the pre-treatment groups. All statistically significant correlation coefficients are marked with a $^\ast $-symbol. The range #x-#y indicates the range of samples that was - due to missing data - minimally and maximally available to calculate the CCs. The CCs of the male groups were generally larger than those of the female groups. For the chosen subset of parameters, VRQOL correlated statistically significantly with age for males with not hoarse sounding voices, $\text{F}_{\mathrm {max}}$ for females with not hoarse sounding voices, $\text{I}_{\mathrm {max}}$ for both female groups and Jit(%) for males with hoarse sounding voices.

**TABLE 5:** Correlation Coefficients

Parameter name	Females #94-#242 FD_01_	Females #74-#207 FD_23_	Males #38-#130 FD_01_	Males #26-#93 FD_23_
GBB	−0.064	0.331*	0.243	0.005
VRQOL	0.058	−0.066	0.311*	−0.082
VHI	−0.020	0.085	−0.271*	0.256
PHQD	0.168	0.040	0.023	−0.009
HADS_A_	0.051	0.168	−0.103	0.077
HADS_D_	0.291*	0.379*	0.075	0.331
$\text{F}_{\mathrm {min}}$	−0.077	0.001	−0.156	0.241*
$\text{F}_{\mathrm {max}}$	−0.189*	−0.080	−0.111	−0.006
$\text{I}_{\mathrm {min}}$	0.125	0.036	0.179*	0.243*
$\text{I}_{\mathrm {max}}$	−0.143*	−0.203*	−0.160	−0.125
DSI	−0.161*	−0.050	−0.218*	−0.269*
MPT	−0.064	0.019	−0.090	−0.065
Jit(%)	0.055	0.147	0.118	0.318*

Spearman correlation coefficients calculated between parameters and subject age. An asterisk indicates a statistically significant correlation. The range #x-#y denotes the range of samples that were minimally and maximally available to calculate the CCs.

The CCs for different parameters vary widely between groups, but never exceed the value of 0.379 (HADS_D_ in hoarse sounding females); i.e. are negligible.

### C. Model Selection and Optimization

After determining the best algorithm and the optimal number of stumps, one AdaBoost model for each of the six group comparisons was created. Each model includes 300 tree stumps (as determined in Methods) and uses all 13 parameters.

For each of these models the feature importance (FI) was calculated and normalized to the parameter with the highest FI. This is illustrated in the supporting information in Fig. S1 for female group comparisons and Fig. S2 for male group comparisons in the partitions (a1), (b1) and (c1) respectively. Additionally, for each group comparison, 13 subsequent models were built. For these models, AUC and ACC are shown in Fig. S1 and Fig. S2 in the partitions (a2), (b2) and (c2). The first model only contains the parameter with the highest FI. The second model contains the parameter with the highest and the parameter with the second highest FI,.... For instance, for the group comparison N_01_ vs. FD_01_ that is depicted in Fig. S1 (a2), the first model only includes $\text{F}_{\mathrm {max}}$, the second model includes $\text{F}_{\mathrm {max}}$ and VHI,….

As can be deduced from Fig. S1 and Fig. S2, the parameters VHI and VRQOL are rated with similar importance by FI. Both are voice related questionnaires that assess the quality of life and hence necessarily not both of them are needed. Scores of both questionnaires are strongly correlated (Spearman correlation factor of -0.83) implying redundancy. Therefore, we propose a reduced set of only four parameters for females and males, namely **VRQOL, I**$_{\mathbf {max}}$**, F**$_{\mathbf {max}}$, and **Jit(%)**.

The removal of **I**$_{\mathbf {max}}$ from this set achieved no difference in average performance (- 0.004 AUC and - 0.002 ACC). However, without $\text{I}_{\mathrm {max}}$ sensitivity and specificity of the group comparison FD_01_ vs. FD_23_ differed more for males (without $\text{I}_{\mathrm {max}}$: 0.145, with $\text{I}_{\mathrm {max}}$: 0.068).

For the final set $\text{I}_{\mathrm {max}}$ was chosen over $\text{F}_{\mathrm {min}}$, although the latter had the higher FI rating in several cases. This was done because, even though all other performance measures were similar for the sets VRQOL, **F**$_{\mathbf {min}}$, $\text{F}_{\mathrm {max}}$, and Jit(%) and VRQOL, **I**$_{\mathbf {max}}$, $\text{F}_{\mathrm {max}}$, and Jit(%), the inclusion of $\text{F}_{\mathrm {min}}$ instead of $\text{I}_{\mathrm {max}}$ lead to a lesser improvement in sensitivity and specificity difference of the group comparison FD_01_ vs. FD_23_ in males (only 0.111).

Also the inclusion of $\text{F}_{\mathrm {min}}$ and $\text{I}_{\mathrm {max}}$ in one set consisting out of five parameters yielded no benefit towards the proposed set (+0.003 AUC and +0.001 ACC).

The set **VHI**, $\text{I}_{\mathrm {max}}$, $\text{F}_{\mathrm {max}}$, and Jit(%) differed not distinctly from the proposed set (+0.002 AUC, - 0.001 ACC).

The set of $\text{F}_{\mathrm {max}}$, VHI, VRQOL, Jit(%) **and I**$_{\mathbf {max}}$ in comparison to the proposed set yielded also no clear difference (-0.004 AUC, - 0.008 ACC).

### D. Comparing Pre- and Post-Treatment

In Table 6, the median values of the parameters from the reduced parameter set and the median absolute deviations are given for the subjects in each pre- and post-treatment-group before and after treatment. The number of available data values for each parameter varied, since for pairwise comparisons any missing numbers needed to be excluded. We decided to use 20 as minimum level for statistical analysis, since 20 is also often considered as the minimum number of subjects that is necessary to get a reliable result in an initial clinical trial [56], [57]. Hence, in the table, parameters that were measured for less than 20 pre-post-treatment pairings are given in brackets. Furthermore, the range of available samples for comparison is given as #x-#y for each row.

**TABLE 6:** Pre-Post-Treatment Comparison

	VRQOL pre	VRQOL post	$\text{I}_{\mathrm {max}}$ pre	$\text{I}_{\mathrm {max}}$ post	$\text{F}_{\mathrm {max}}$ pre	$\text{F}_{\mathrm {max}}$ post	Jit(%) pre	Jit(%) post
Females median/median absolute deviation								
PrePost_01/01_ #26-#46	62.5/20	82* /18	89.5/3.5	89/4.5	552.5/106.5	608.5/158.5	0.160/0.05	0.140/0.05
PrePost_23/01_ #20-#37	59.5/17.5	88.5* /8.5	90/6	91* /6	428/85	535* /111	0.220/0.150	0.170* /0.06
PrePost_23/23_ #18-#31	(42/15)	(47/13)	84/5.5	82/4.5	420/67	384/82	0.670/0.590	0.90/0.700
Males median/median absolute deviation								
PrePost_01/01_ #17-#29	(75/12)	(87* /8)	92/5	92/5	330/79	370/119	0.120/0.050	0.160/0.090
PrePost_23/01_ #4-#10	(80/5)	(87.5/5)	(89.5/3.5)	(93.5* /3.5)	(445/109)	(395/96.5)	(0.145/0.055)	(0.165/0.090)
PrePost_23/23_ #5-#9	(75/10)	(72/5)	(85/7)	(84/5)	(359/75)	(308/146)	(0.160/0.030)	(0.925/0.770)

Median values of the parameters from the reduced parameter set for all pre-post-treatment group comparisons. If one parameter improved statistically significantly this is marked by a *-symbol at the post median-value of the parameter. If a pre-post-pairing included less than 20 parameters, the median values in the table are set in brackets. The range ”#x-#y” denotes the minimal and maximal number of available samples for each row.

## IV. Discussion

### A. Summary

VRQOL reflects mostly the differences between N_01_ and FD_01_ as well as N_01_ and FD_23_. Jit(%) reflects the differences between FD_01_ and FD_23_. $\text{I}_{\mathrm {max}}$ contributes mainly to a more balanced classification between the groups of disordered males. $\text{F}_{\mathrm {max}}$ is the parameter with the highest FI on average. All four parameters are reflecting treatment outcomes in pre-post-treatment comparisons with statistical significance for females.

### B. Influence of Subject Age

For the final parameter set (VRQOL, $\text{I}_{\mathrm {max}}$, $\text{F}_{\mathrm {max}}$, Jit(%)) five correlations were statistically significant (Table 5). However, two of these correlations were barely over the 0.3 limit for negligibility as proposed by Mukaka [49] and three were below this limit. Hence, the influence of age is seen as negligible.

### C. Model Selection and Optimization

For the final set of parameters VRQOL, $\text{I}_{\mathrm {max}}$, $\text{F}_{\mathrm {max}}$ and Jit(%) were chosen. Different questionnaire scores were given a rather high FI in the models. VRQOL and VHI were the overall best parameters in separating healthy subjects (N_01_) from both functional dysphonia groups (FD_01_, FD_23_). This is expected, since both are self-assessment questionnaires that capture if the subject feels somehow impaired by its voice. Since FD is such a broad term including many different symptoms and causes [16], the individually perceived impairment of the voice is lastly the variable that unifies all manifestations of FD. VRQOL and VHI scores have shown to be strongly associated before [58] and are also highly correlated in our data; therefore the inclusion of both brings no benefit for classification. We decided to only include VRQOL because it consists of fewer questions than VHI and is therefore more easily to collect. Furthermore VRQOL has shown evidence of longitudinal validity [59]. Hence it is expected to also accurately reflect the course of treatment.

Of all parameters, Jit(%), $\text{F}_{\mathrm {min}}$ and $\text{I}_{\mathrm {max}}$ achieved highest FI ratings for separating FD_01_ and FD_23_ in males (see Fig. S2). Whilst the addition of neither $\text{F}_{\mathrm {min}}$, nor $\text{I}_{\mathrm {max}}$ to the parameter set yielded a distinct increase in AUC or ACC, both contributed by decreasing the difference between sensitivity and specificity, mainly for the male group comparison FD_01_ vs. FD_23_. Males have a lower speaking fundamental frequency than females [60] and whilst $\text{F}_{\mathrm {max}}$ was more important to identify voice disorder in females (see Fig. S1 and S2), maximum intensity ($\text{I}_{\mathrm {max}}$) and minimum frequency ($\text{F}_{\mathrm {min}}$) were more important in males. However, the addition of only $\text{I}_{\mathrm {max}}$ to VRQOL, $\text{F}_{\mathrm {max}}$ and Jit(%) yielded a greater improvement in comparison to the addition of only $\text{F}_{\mathrm {min}}$ or the addition of both.

In literature, values of Jit(%) for healthy subjects phonating the vowel /a/ vary. Values of 0.25% for females and males are given for clinical data [61], but also values as high as 0.53% for younger and 0.84% for older males are considered as healthy [62]. Such differences may be due to differences in recording settings. For example, the sampling rate has an influence on some parameters as it was illustrated for Jit(%) (albeit on Glottal area waveform-based signals) [41]. Nevertheless, increased period perturbation i.e. increased Jitter indicates a disordered voice [60], [61] if the recording conditions do not change, as it was the case for this study.

Since Jit(%) is a cycle-based parameter, it relies on the correct detection of phonation cycles and it is known that Jitter measures lack robustness towards aperiodic signals, as seen in highly hoarse voices [63]. Jit(%) is naturally dependent on the algorithm that was chosen for cycle detection. If cycle detection fails, as it may be the case for more aperiodic voices, the detected cycles may be even more aperiodic than the actual ones, artificially increasing Jit(%). Therefore it is to be expected, that the underlying cycle detection algorithm may play a more important role in data separation than Jit(%) itself, but still the calculated value of Jit(%) can reflect better than any other of the investigated parameters if a voice is hoarse or not.

Adding $\text{F}_{\mathrm {max}}$ as fourth parameter yields the greatest improvement in performance for almost all comparisons. For the generation of a frequency as high as possible the vocal folds have to oscillate as fast as possible. Therefore, it seems natural that if a subject has no voice disorder it can produce higher frequencies. It has also been shown before that patients after successful treatment of a voice disorder are able to produce a higher fundamental frequency [64] and that maximum fundamental frequency is an important feature for judging voice quality [37].

Overall, a great amount of parameters can be used to describe voice pathology [65]. Collecting and applying all of them in a clinical setting would not be possible. Currently no standards for parameter collection exist in clinics. Therefore, to enhance clinical exchange and standardize treatments, parameters need to be found that best describe different voice pathologies.

VRQOL, $\text{I}_{\mathrm {max}}$, $\text{F}_{\mathrm {max}}$ and Jit(%) describe different features and are important for the separation between FD and healthy groups. However, no single parameter was able to differentiate between all groups. Also the addition of more than the four proposed parameters did not significantly improve the overall performance of the group separation tasks. This shows that approaches to classify clinical data need to be multidimensional, but the inclusion of a too large number of parameters may not result in better performance.

### D. Comparing Pre- and Post-Treatment Groups

As it can be derived from Table 6, for the pre-post-treatment comparison **“PrePost**$_{\mathbf {01/01}}$” most parameters from the reduced dataset improved on average for females and males. VRQOL assesses patient quality of life and was the only parameter from the reduced set to improve statistically significantly. This implies that the treatment on average improved the well-being of PrePost_01/01_ patients. However, the voice of these patients was not hoarse and thus did also not change during treatment, as it is indicated by the constant good voice quality ratings of the clinicians (RBH-H = 0,1). Since the objective parameters $\text{F}_{\mathrm {max}}$, $\text{I}_{\mathrm {max}}$ and Jit(%) did not improve statistically significantly, they may be less important for identifying not-hoarseness-related FD. Noteworthy, these three objective parameters still improved on average for females and, in case of $\text{F}_{\mathrm {max}}$, also for males.

For females, in the pre-post-treatment comparison **“PrePost**$_{\mathbf {23/01}}$”, all parameters improved statistically significantly. This is expected, as the decrease in RBH-H from pre- to post-treatment indicates an improvement in voice quality. The increase in $\text{I}_{\mathrm {max}}$ was only minor, yet still statistically significant, since the increase was consistent for most patients (p $=0.0066$). However, for male subjects only $\text{I}_{\mathrm {max}}$ improved statistically significantly. The average value for $\text{F}_{\mathrm {max}}$ and Jit(%) even worsened. Albeit this may easily accounted by the very low sample size, since for $\text{F}_{\mathrm {max}}$ only 10 and for Jit(%) only 8 male pre-post-treatment observations were available.

For the last pre-post-treatment comparison **“PrePost**$_{\mathbf {23/23}}$” none of the parameters improved statistically significantly and some even worsened on average (e.g. $\text{F}_{\mathrm {max}}$ for females). The continuously high RBH-H for this group comparison, even after treatment, indicates no improvement in voice quality and hence a not yet successful treatment or failure of the treatment. Therefore, it seems expectable that the parameters do not improve.

Considering all pre-post-treatment comparisons, VRQOL, $\text{I}_{\mathrm {max}}$, $\text{F}_{\mathrm {max}}$ and Jit(%) in general reflect the treatment outcomes as they show improvement for successful treatments; i.e. RBH-H improves. On the other hand, these parameters do not improve for unsuccessful treatments; i.e. high RBH-H before and after treatment. For the case of continuously low RBH-H only VRQOL improves statistically significantly on average, indicating that the subjective wellbeing of the patient regarding his voice increased. Since no hoarseness related voice disorder was present from the beginning for these patients, it is also expected that parameters reflecting hoarseness, i.e. Jit(%) and partially $\text{I}_{\mathrm {max}}$ and $\text{F}_{\mathrm {max}}$, show no statistically significant improvement.

### E. Shortcomings

No additional validation set was used after cross-validation. This was done, because performance measures varied considerably depending on the testing partition. Also performance varied depending on partitioning of the model. For this reason average performance of ten ten-fold cross validated models with different partitioning for all testing partitions was reported instead.

Since all data investigated in this work was recorded under clinical conditions, the typical hindrances of clinical settings (not optimal recording conditions, missing values, changing examiners) apply to this work. Also, time between pre- and post-treatment varies between one week and one year, depending on patient-specific recommendations by our clinicians.

Due to the different age ranges of the pre-treatment groups and the healthy group, the results of this study may be influenced by subject age. An influence of subject age for different signal types and voice parameters is well documented in the literature [66]–[68]. Based on some parameters even age prediction is possible [69]. However, besides Jit(%), different parameters were used in this work and, as the investigation of the correlation between subject age and parameter values imply, the influence of age is negligible for our data. Nevertheless, correlation only captures linear associations between variables, more complex, nonlinear relations are possible.

Validity of parameters varies on a spectrum and no parameter is 100% reliable. Further, different questionnaires and objective parameters exist in different countries and it is possible that, by using those questionnaires, an even better separation of groups would be achievable. Objectively calculable parameters may vary depending on its software implementation [63], [70]. This is especially important since depending on the method of cycle detection the usefulness of Jit(%) for data separation may vary.

## V. Conclusion

Scientific Outcomes: In this study, we show that only a small set consisting of four parameters (VRQOL, $\text{I}_{\mathrm {max}}$, $\text{F}_{\mathrm {max}}$ and Jit(%)) is sufficient to differentiate between healthy subjects and patients with diagnosis of functional dysphonia; i.e. these parameters reflect FD induced impairments. We confirm boosted stumps as a reliable tool for classification of incomplete clinical datasets and also show that subject age is negligible for the considered parameters.

Clinical Outcomes: The final set of four parameters also reflects treatment outcome for FD and perceivable hoarse voices (success or failure). For FD without hoarse voices, the improvement of only VRQOL but not the objective parameters demonstrated treatment success, as perceived on a subjective level by the patient. Therefore, VRQOL describes general treatment outcome whilst $\text{I}_{\mathrm {max}}$, $\text{F}_{\mathrm {max}}$ and Jit(%) describe treatment outcome for patients with hoarse voices.

This study furthermore confirms that multidimensional approaches are needed for the assessment of clinical datasets since single parameters are not sufficient for data separation. Therefore, by finding the best and most relevant parameters, in future a functioning set of objective tools could be created that improves and accelerates assessment and therapy of voice disorders.
